# The Role of Nucleoporin Elys in Nuclear Pore Complex Assembly and Regulation of Genome Architecture

**DOI:** 10.3390/ijms21249475

**Published:** 2020-12-13

**Authors:** Yuri Y. Shevelyov

**Affiliations:** Department of Molecular Genetics of Cell, Institute of Molecular Genetics of National Research Centre “Kurchatov Institute”, 123182 Moscow, Russia; shevelev@img.ras.ru; Tel.: +7-499-196-0809

**Keywords:** nucleoporin, Elys, Mel-28, NPC, nuclear envelope, genome architecture, chromatin

## Abstract

For a long time, the nuclear lamina was thought to be the sole scaffold for the attachment of chromosomes to the nuclear envelope (NE) in metazoans. However, accumulating evidence indicates that nuclear pore complexes (NPCs) comprised of nucleoporins (Nups) participate in this process as well. One of the Nups, Elys, initiates NPC reassembly at the end of mitosis. Elys directly binds the decondensing chromatin and interacts with the Nup107–160 subcomplex of NPCs, thus serving as a seeding point for the subsequent recruitment of other NPC subcomplexes and connecting chromatin with the re-forming NE. Recent studies also uncovered the important functions of Elys during interphase where it interacts with chromatin and affects its compactness. Therefore, Elys seems to be one of the key Nups regulating chromatin organization. This review summarizes the current state of our knowledge about the participation of Elys in the post-mitotic NPC reassembly as well as the role that Elys and other Nups play in the maintenance of genome architecture.

## 1. Introduction

In eukaryotic cells, the nuclear-cytoplasmic transport of macromolecules occurs through channels in the nuclear envelope (NE) formed by nuclear pore complexes (NPCs). NPCs are large multiprotein structures consisting of ~30 nucleoporins (Nups) each represented by 8–32 copies [1]. Apart from transport function, Nups interact with chromatin, thus affecting genome architecture and gene expression [2,3,4,5,6,7,8,9,10,11,12,13,14,15,16,17,18]. While in yeast, the recruitment of genes to the NPCs causes their activation and is accompanied by the emergence of transcriptional memory [19,20], in higher eukaryotes the picture is more complicated. In metazoans, some Nups are localized not only at the NE, but also in the nucleoplasm [21,22,23]. Moreover, while nucleoplasmic Nups bind to active genes promoting their transcription, the constituents of NPCs at the NE mostly interact with the silent genes, or those poised for activation [8,9,10,12,13,15,16,17,18]. In a similar fashion to yeast, the transcriptional memory was revealed in *Drosophila* and mammals. In mammals, this mechanism may be exemplified by the interaction of interferon-inducible genes with the nucleoplasmic Nups [24]. In contrast in *Drosophila*, ecdysone-inducible genes interact with the NPC-linked Nups [16].

Numerous data suggest that peripheral chromatin interacts with both components of the NE–the nuclear lamina (NL) and NPCs. The NL is a meshwork of lamins and lamin-associated proteins which lines the inner nuclear membrane [25]. A decade ago, lamina-associated chromosomal domains (LADs) were revealed in *Drosophila*, mammals and nematode [26,27,28,29,30]. It was shown that either artificial disruption or a naturally occurring reduction of specific components of the NL resulted in chromatin relocalization from the NE to the nuclear interior, thus indicating that peripheral chromatin is attached to the NL [31,32,33,34,35,36,37,38,39,40,41,42].

Several findings indicate that NPCs may also be involved in the anchorage of chromosomes to the NE. In *C*. *elegans*, the single X chromosome in males has a more peripheral position in the nucleus than the two X chromosomes in hermaphrodites and this spatial organization correlates with more pronounced interactions of the single X chromosome with one of the Nups [43]. Similarly, the single X chromosome in *Drosophila* SL-2 cells appears to be more strongly bound with Nups, such as Megator (Mtor) and Nup153, and is located closer to the NE than the autosomes. This proximal position of the X chromosome relative to the NE becomes more distal upon Nup153 depletion [10]. Furthermore, depletion of Nup153 in mouse embryonic stem cells (mESCs) leads to several Nup153-target loci being located further from the NE [14]. Additionally, depletion of Nup98 in *Drosophila* S2 cells results in the relocalization of two ecdysone-inducible genes from the NE to the nuclear interior [16]. Collectively, these findings support the model whereby the interactions of the genome not only with the NL, but also with the NPCs are important for determining the intranuclear positioning of chromosomes.

The involvement of Nups in these interactions with chromatin arouses a particular interest in Elys (embryonic large molecule derived from yolk sac), also known as Mel-28 (maternal effect lethal-28) or AHCTF1 (AT-hook containing transcription factor 1), as the Nup that can bind chromatin directly. Although several recently published reviews have described participation of Nups in chromatin organization [44,45,46,47], these papers did not focus on Elys. Here, I discuss the role that Elys plays in the post-mitotic NPC reassembly and in establishing and maintenance of overall genome architecture.

## 2. Role of Elys in Post-Mitotic Reassembly of NPCs and Other NE Components

**Figure 1:**
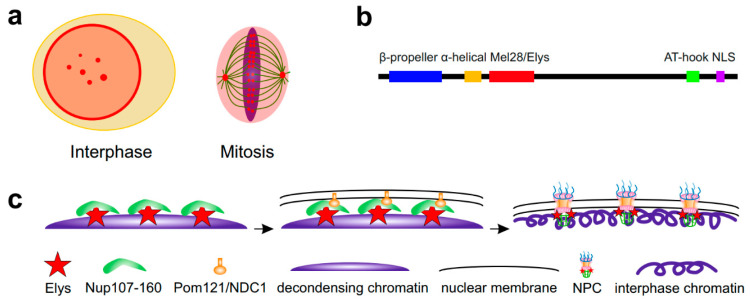
Schematic showing Elys localization and participation in post-mitotic NPC reassembly. (**a**) During interphase, Elys (red) is mainly localized at the NE and in GLFG bodies located in the nuclear interior. During mitosis, Elys is mainly localized around spindle poles and on kinetochores. (**b**) Elys domain structure according to [57]. (**c**) In anaphase, Elys binds to the decondensing chromatin and recruits Nup107–160 NPC subcomplex. Next, transmembrane Nups such as Pom121 and NDC1 link these structures with the re-forming nuclear membranes. Then, the remaining NPC components are recruited resulting in the formation of complete NPCs which penetrate nuclear membranes and are attached to the interphase chromosomes.

Elys was initially identified as an AT-hook-containing transcription factor which is strongly expressed in embryonic hematopoietic tissues and more weakly in other mice tissues, localizing to both the nucleus and cytoplasm [48]. However, later studies did not support the presence of Elys in the cytoplasm. During interphase, Elys was shown to colocalize with the NPCs at the NE in different organisms including mammals, *Xenopus*, nematode and *Drosophila* [49,50,51,52,53,54] (Figure 1a, left panel). Accordingly, Elys contains the N-terminal β-propeller and α-helical domains, which are both responsible for its association with the Nup107–160 subcomplex of NPC [55,56] (Figure 1b). In addition to its localization at the NE, Elys is also diffusely distributed in the nuclear interior [51] concentrating in the dot-like structures known as GLFG bodies [21,23]. During mitosis, Elys is located at the surface of chromatin masses as well as residing at kinetochores and spindle poles [49,50,51,56,57] (Figure 1a, right panel). In nematode and mammals, the localization of Elys at kinetochores is necessary for mitosis to proceed correctly [49,50,51]. However, in *Drosophila*, Elys was not observed to reside at the kinetochores [54]. Interestingly, the entire Nup107–160 subcomplex also resides at kinetochores during mitosis in mammals [58], but not in *Drosophila* [59]. Therefore, although it was not shown directly, Elys, together with the Nup107/160 subcomplex, seems to be dispensable for mitotic chromosome segregation in *Drosophila*.

The process of NPC reassembly at the end of mitosis consists of sequential steps of recruitment of NPC subcomplexes to the re-forming NE. It is initiated in anaphase by Elys binding to the decondensing chromatin via the AT-hook and another C-terminal domain [60], followed by Elys-mediated recruitment of the Nup107–160 subcomplex of NPC [51,52,60,61,62,63] (Figure 1c). Field emission scanning electron microscopy confirms the colocalization of Elys with the Nup107–160 subcomplex on chromatin “seeding” sites during NPC reassembly [64]. Consistent with Elys targeting of Nup107–160 subcomplex to chromatin, structural studies have shown that Elys is directly associated with the Nup160 subunit of this subcomplex [65,66]. During the next step of NPC reassembly, the transmembrane Nups such as Pom121 and NDC1, which are already integrated in the inner nuclear membrane, bind to the Nup107–160 subcomplex; together, this complex recruits other components of the NPC to this site [60,67,68]. Depletion of Elys results in the failure of NPCs to localize at the NE [50,51,52,63], thus indicating that Elys is strictly required for NPC reassembly at the end of mitosis. However, besides post-mitotic reassembly, NPCs may be incorporated in the NE during interphase via an Elys-independent pathway [63,69]. Interestingly, RNA is also implicated in the post-mitotic NPC reassembly via Elys, although the details of this process are not clear [70].

Despite the requirement of Elys for the post-mitotic reassembly of NPCs, depletion of Elys does not notably impair the incorporation of lamins in the NL in mammals [51,71], although some relocalization of B1 and B2 lamins to the cytoplasm was reported [72]. At the same time, Elys depletion correlates with a drastic disruption of the NL in *Drosophila* salivary gland cells [54]. However, the latter effect may be the consequence of cell death, rather than the requirement of Elys for the post-mitotic reassembly of lamins, since depletion of Elys was shown to induce apoptosis in several cell types [54,73]. Another important component of the NL is lamin-B-receptor (LBR), which is an integral membrane protein of the NE and is associated with lamins [74,75]. In mammals, lack of Elys partially displaces LBR from the NE to the cytoplasm in a phosphorylation-dependent manner during interphase [71] and perturbs the recruitment of LBR to the re-forming NE during telophase [76].

## 3. Genome-Wide Interactions of Elys and Other Nups

Using DamID [77] or chromatin immunoprecipitation (ChIP) techniques, genomic regions interacting with various Nups, including Elys, were recently mapped in metazoan organisms. First, the Nup93-interacting regions were identified in HeLa cells [6]. Surprisingly, unlike yeast, where recruitment of genes to NPCs mostly correlates with gene activation [2,3,4,5,11,19,20], Nup93-interacting regions in mammals were enriched in repressive histone modifications [6]. Further mapping of Nup98-, Nup50-, Nup62-, Nup153- and Mtor-interacting regions in the *Drosophila* genome has revealed that these dynamic Nups [22] largely interact with active genes located in the interior part of a nucleus [8,9,10]. Importantly, Kalverda et al. were able to separate interactions of Nup98 with *Drosophila* genome which occur via NPCs located at the NE from those occurring in the nuclear interior [9]. It appeared that NPC-linked Nup98 mainly interacts with short ~2 kb genome regions embedded in inactive chromatin, whereas the nucleoplasmic fraction of Nup98 predominantly interacts with broader genome domains carrying a high level of histone acetylation and transcriptionally active genes [9]. Moreover, NPC-linked domains comprise only ~20% of all Nup98-interacting domains [9]. Interactions with other dynamic Nups also seem to occur predominantly away from the NE [8,10]. These data indicate that most genome interactions with dynamic Nups occur in the nuclear interior, far from NPCs. Further high-resolution ChIP-seq mapping of Nup98-bound regions in human and *Drosophila* genomes has shown that peaks of Nup98 binding mainly coincide with promoters and, to a lesser extent, with enhancers of developmentally regulated or inducible genes [12,16]. The association of Nup98 mainly with promoters was also confirmed by the DamID approach in human cells [78]. Moreover, Nup107-interacting regions in *Drosophila* also mainly correspond to promoters or to other active chromatin regions [18].

However, in agreement with the earlier results in human cells [6], ChIP-seq mapping of Nup93-genome interactions in *Drosophila* revealed that this Nup is mainly associated with inactive chromatin regions silenced by Polycomb (Pc) group proteins that are enriched with H3K27me3 histone modifications [16,18]. In particular, some Nup93 interacting sites colocalize with the Pc response elements (PREs) [18], which are necessary for Pc-mediated repression of developmental genes. Moreover, Nup93 was coimmunoprecipitated with Pc from protein extracts of S2 cells [18]. At the same time, although less frequently, Nup93 interacts with H3K27 acetylated regions [18]. The latter finding is in line with the results of Ibarra et al., who found that Nup93-interacting regions in the human genome colocalize with super enhancers which are marked by peaks of H3K27 acetylation [15]. As the stable components of the inner-ring sub-complex of NPC [22,79], Nup93 is minimally present in the nucleoplasm. Accordingly, Nup93-bound loci were localized by FISH mainly at the NE [15,18]. Therefore, Nup93 binding to either inactive or active chromatin in fact represents binding to the NPCs at the NE.

Nup153 is a component of the nuclear basket of NPCs [79] and thus it may be tightly associated with peripheral chromatin. However, as a dynamic Nup [22], it may also interact with chromatin in the nuclear interior. Mapping of Nup153-interacting regions in mammalian genomes by DamID or ChIP-seq has shown that, depending on cell type, Nup153 may interact with the active H3K27 acetylated chromatin of super enhancers [15], with the inactive Pc-repressed chromatin of developmental genes [14], with the promoters and 3′-ends of Sox2-regulated genes [80], or with active and inactive promoters, as well as with enhancers and boundaries between topologically associating domains (TADs) [81]. Nup153-genome interactions take place both at the NE and in the nucleoplasm. Consistent with its enrichment at TAD boundaries, Nup153 was shown to physically associate with cohesin and CCCTC-binding factor (CTCF) [81], the proteins which participate in TAD formation in mammals through the loop extrusion mechanism [82,83,84]. However, the impact of Nup153 (or other Nups) on TAD formation has not yet been examined.

**Figure 2:**
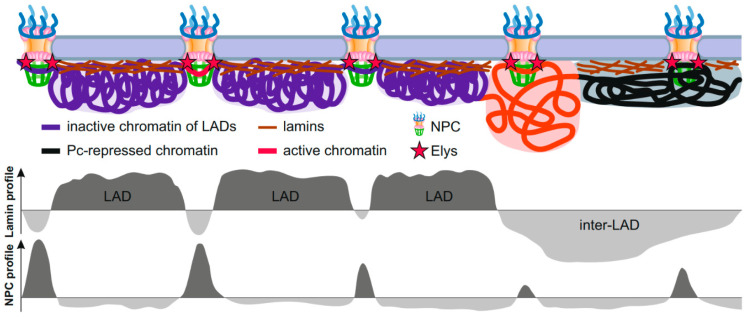
Schematic showing interactions of peripheral chromatin with the NL and NPCs. Interphase chromosomes are attached to the NL in LADs and, simultaneously, are attached to the NPCs (probably via Elys) in short genomic regions embedded either in LADs (violet), or in Pc domains (black), or in active chromatin domains (red). Putative profiles of genome interactions with lamin or NPCs are shown below.

Interestingly, the summit in the genome-averaged NPC-linked Nup98 DamID profile exactly corresponds to the dip in the average DamID profile of lamin in *Drosophila* [9]. This observation was recapitulated by ChIP-seq [18], thus indicating that some of the NPC-interacting regions appear to be embedded in LADs. These findings support the model that extended chromatin fiber is alternately attached to either the NL (via LADs) or to the NPCs (via NPC-linked sites), with both types of genome regions being tightly interspersed [18,85] (Figure 2). As the NPC-linked sites mostly contain promoters and enhancers of a subset of developmentally regulated or inducible genes [9,15,16,18], this model refines previously obtained data that the activated promoters of tissue-specific genes lose an association with the NL, whereas the bodies of these genes retain contacts with the NL [27,86,87,88]. Moreover, an improved bioinformatic analysis of high throughput chromosome conformation capture (Hi-C) [89] coupled with the Lamin-DamID data in mammalian cells allowed to identify the dips in the LAD profile correlated with the short “islands” of H3K27 acetylation, as well as with the positions of active promoters and enhancers of low-expressed genes which are frequently bound with cohesin and CTCF [90]. Gozalo et al. supposed that these dips in LAD profile correspond to genomic sites attached to the NPCs [18].

It should be noted that the majority of the above-mentioned Nup-genome interactions seem to be indirect. They may be mediated either by Elys, which is capable of binding to chromatin directly [60,91,92], or by other chromatin-bound protein complexes unrelated to NPCs (see below). Elys is the only Nup containing both DNA- and chromatin-binding domains, and due to these features, it may directly interact with chromatin. The association of Elys with chromatin was first revealed in experiments with *Xenopus* sperm chromatin [52,61]. The presence of the AT-hook motif [48] implies the ability of Elys to bind to AT-rich DNA sequences. Indeed, the antibiotic Distamycin A, which binds to the minor groove of AT-rich regions of DNA, notably impairs the association of Elys with chromatin [60]. In addition, mutations in the non-canonical AT-hook motifs of *Drosophila* Elys decrease its DNA binding ability as assessed by electrophoretic mobility shift assay [54]. Surprisingly, apart from AT-hook, the most C-terminal domain of Elys also appears to be responsible for chromatin binding [56,60,92] (Figure 1b). Furthermore, it was shown that the C-terminal domain of Elys, including the AT-hook, directly binds nucleosomes and H2A/H2B histone dimers [91,92]. This binding occurs with the acidic patch of H2A/H2B dimers [93,94].

Elys binding sites in the *Drosophila* genome were recently mapped by ChIP-seq in the brains of third instar larvae [16] as well as in embryonic S2 cells [18]. Importantly, the overwhelming majority of Nup98 and Nup93 ChIP-seq peaks in larval brains, or Nup107 and Nup93 ChIP-seq peaks in S2 cells were not intersected, but, instead, strongly overlapped with the peaks of Elys [16,18]. Moreover, consistent with the ability of Elys to bind chromatin, Elys ChIP-seq peaks were most robust and prominent when compared with Nup98, Nup107, or Nup93 peaks [16,18]. Therefore, it is tempting to suggest that Elys mediates binding of NPCs with both active (marked by Nup98 or Nup107) and inactive (marked by Nup93) genomic sites, as well as participating in the interactions of Nups with active chromatin in the nuclear interior.

Interestingly, Elys may not only bind intranuclear loci, but also target them to NPCs for gene gating. In particular, in colon cancer cells, Elys binds the oncogenic super enhancer and directs it to the NPC-linked *MYC* gene, which drastically increases nuclear export of *MYC* mRNAs [95].

## 4. Influence of Elys and Other Nups on Gene Expression

Given the plethora of Nup-genome interactions, the question arises as to whether they affect gene expression. Several groups have found that interactions of dynamic Nups with genes positioned in the nucleoplasm facilitate the expression of some of these genes [8,9,10,12]. For example, depletion of Nup98 in *Drosophila* Kc167 cells resulted in the downregulation of a subset of genes which, more strongly than other genes, interact with the nucleoplasmic Nup98, but interact at a similar level with NPC-linked Nup98. In addition, vice versa, overexpression of Nup98 led to the increased expression of genes interacting with Nup98 preferentially in the nucleoplasm but not at NPCs [9]. In a similar way, an overexpression of Nup98 in human neural progenitor cells increased transcription of 12 out of 24 randomly selected Nup98-interacted genes responsible for neural development [12].

For a long time, the mechanism of gene activation by nucleoplasmic Nups remained enigmatic. In attempts to clarify this issue, Pascual-Garcia et al. found that ~40% of Nup98 binding sites in S2 cells colocalize with the sites occupied by the methyl binding domain-related 2 (MBD-R2) subunit of the nonspecific lethal (NSL) complex, as well as by H3K4-specific histone methyltransferase Trithorax (Trx) [13]. Moreover, the authors have shown that Nup98 is physically associated with both MBD-R2 and Trx, and may be recruited to chromatin by MBD-R2 [13]. An earlier whole-genome ChIP-seq analysis has revealed that the NSL complex binds to the majority of housekeeping gene promoters and, by introducing H4K16 acetylation, is required for their transcriptional activation [96,97]. Therefore, MBD-R2 may be responsible for Nup98 targeting of active promoters. However, it remained unclear how the presence of Nup98 on a promoter may stimulate gene expression. It is unlikely that this occurs via NSL or Trx activities, since Nup98 depletion, while downregulated *Ubx* transcription, neither affected H4K16 acetylation, nor H3K4 methylation of the *Ubx* gene promoter [13].

**Figure 3:**
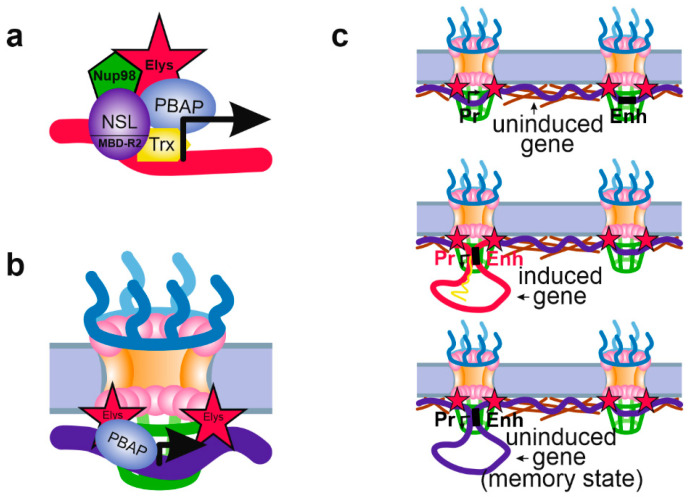
Schematic showing various mechanisms of Nup influence on chromatin state. (**a**) Nup98 may be recruited to the active promoters, positioned in the nuclear interior, by the NSL complex (via MBD-R2 subunit) or by Trx. Nup98 may then recruit Elys associated with PBAP complex, although this was not shown directly. (**b**) The NPC-linked promoters of tissue-specific or inducible genes may be prepared for further activation by PBAP complex associated with Elys. PBAP remodels chromatin making it more “open” and inducible. (**c**) Attachment to the NPCs of a promoter and enhancer of an inducible gene may facilitate their communications. Without induction, promoter and corresponding enhancer may be attached to different NPCs (upper panel). Upon induction, promoter and enhancer become linked together thus ensuring active transcription (middle panel). After removal of the inductor, promoter and enhancer remain in contact with each other and with the NPC (the transcriptional memory state), which allows transcription to be more rapidly activated upon repeated induction (low panel). (**a**–**c**) Designations are the same as in Figure 2.

Recently, the work of the same group has shed light on this mechanism. The authors have shown that artificial tethering of Nup62 or Sec13 to genomic loci leads to the recruitment of Elys to these sites correlated with decompaction of their chromatin. This was accompanied by transcriptional activation of some of these loci [17]. Additionally, the authors were able to coprecipitate Elys and Sec13 together with the SWI/SNF ATP-dependent chromatin remodeling complex PBAP (Polybromo-containing Brahma-associated proteins) [98] from *Drosophila* S2 cell extracts. Earlier, the association of Elys with the SWI/SNF subunits was detected in *C*. *elegans* [99]. PBAP is able to release histone octamers from gene promoters, thus making these regions more accessible for binding with transcription factors and with RNA polymerase II [17]. Therefore, binding of Elys and/or Sec13 induces chromatin opening which may further facilitate gene transcription. Interestingly, based on micrococcal nuclease digestion assay, the authors detected general increase in chromatin compaction upon Elys depletion [17]. It seems plausible that the general chromatin compaction accompanied by a reduction of nucleus size, that was revealed upon Elys loss in the previous reports [52,61,70,72], may be the result of disturbed targeting of PBAP by Elys to active chromatin. Given that artificial tethering of LacI-Nup98 to a genomic site carrying LacO array led to the recruitment to this site of other Nups, including Elys [68], it is not surprising that almost all Nup98-binding regions in *Drosophila* larvae brains coincided with Elys-binding regions [16]. According to results of Kalverda et al. [9], ~80% of Nup98 sites represent interactions of active chromatin with this Nup in the nuclear interior. It therefore seems likely that Nup98 may recruit Elys associated with the PBAP complex [17] to the target promoters, thus creating a more favorable environment for the transcription of these genes (Figure 3a). The mechanism of transcriptional facilitation by Elys linked to PBAP may function both in the nuclear interior and at the NPCs (Figure 3b).

Similarly to yeast, some of the gene promoters occupied by Nups in metazoans demonstrate transcriptional memory—a more rapid and strong induction of transcription in response to repeated external signals [3,16,19,24]. Such a mechanism may operate both at the NE and in the nucleoplasm. For example, the recruitment of Nup98 to the promoters of several interferon-y-responsive genes after 48 h of interferon induction was shown to be required for the efficient transcriptional reactivation of these genes in HeLa cells. Nup98 binding to these promoters, occurring in the nucleoplasm, is accompanied by the appearance of paused RNA polymerase II and H3K4 methylation at the promoters, both of which are thought to ensure their more effective transcriptional reactivation [24]. Further insight into this mechanism was performed by Pascual-Garcia et al. [16] on *Drosophila*. The authors have found that the binding of Nup98 with the promoters and enhancers of ecdysone-inducible genes mediates the formation of a chromatin loop between these regulatory elements. This spatial connection of enhancer and promoter is necessary for rapid reactivation of expression of these genes upon repeated treatment with ecdysone. Moreover, Nup98 was found to be associated with the architectural proteins CP190, CTCF and Su(Hw) (suppressor of Hairy-wing) which may also participate in the looping out of chromatin. These findings support a mechanistic model whereby NPC-anchored enhancer-promoter looping of poised genes, preserved after removal of an inductor, may facilitate more rapid reactivation of these genes in response to external stimuli [16] (Figure 3c). A similar model was recently suggested to explain rapid activation of immediate early gene transcription in response to an induction by epidermal growth factor in HeLa cells [81]. In this case, the NPC-linked Nup153 controls binding of CTCF and cohesin with the promoter and enhancer of uninduced gene, leading to RNA polymerase II pausing on its promoter. Upon induction, CTCF and cohesin are released from the enhancer which results in alterations of local chromatin architecture and in redirection of locus to the nuclear interior.

## 5. Conclusions and Outlooks

It seems likely that the sites on decondensing chromatin, to which Elys is attached during NPC reassembly at the end of mitosis, remain bound with Elys (and with NPCs via Elys) in interphase. The number of nuclear pores as well as the number of NPCs incorporated in the NE doubles during interphase [69,100]. Although NPC incorporation in the NE during interphase occurs through an Elys-independent pathway [63], Elys becomes associated with the newly incorporated NPCs as well. Moreover, it looks like that these newly incorporated NPCs also interact with the genome via Elys. This idea is supported by the findings that (i) almost all binding sites of Nup98 are overlapped with that of Elys [16], and (ii) NPC-linked Nup98 interacts with the ~2 kb genomic sites [9], half of which originated due to Elys post-mitotic binding.

Since the nucleoplasmic fraction of Nups interacts with the broad genomic domains enriched with active chromatin marks, whereas NPC-linked Nups interact with the short genomic sites embedded in LADs, or in active chromatin regions, or in Pc domains [9,18], it is tempting to speculate that mechanisms of Nup–genome interactions may be different in these two locations. Whereas genomic regions, interacting with Nup98 or other dynamic Nups, including Elys, in the nuclear interior, may represent the result of targeting of these Nups to the active, H3K27 acetylated chromatin, the short genomic sites attached to NPCs may be selected by sequence-specific DNA recognition by Elys. If this is the case, NPC-linked sites are expected to be rather conservative among cell types, whereas genome regions interacting with nucleoplasmic Nups should be variable during development. The latter idea is supported by the results of Liang et al., who have found that the pattern of Nup98-genome interactions is drastically changed upon differentiation of human embryonic stem cells to neural progenitor cells [12]. The hypothesized sequence-specific mode of Elys-mediated NPC binding could explain promiscuous interactions of NPCs with various chromatin types. Upon this scenario, Nups within NPCs may additionally stabilize Elys binding with the genome. Nup93 or Nup153 may associate with Pc group proteins [14,18] and thereby stabilize interactions of NPCs with genomic regions silenced by Pc. In the cell types, where those regions are in the active state, their interactions with NPCs may be stabilized, for example, by the Trx, associated with NPCs via Nup98 [13]. However, the supposed sequence-specific mode of Elys binding still awaits verification.

In summary, Elys is the major player in the post-mitotic reassembly of NPCs. Additionally, it partially affects the restoration of other components of the NE at the end of mitosis. Apart from these functions, Elys likely determines the sites by which the genome is attached to the NPCs. A subset of these interactions results in transcriptional memory—more rapid reactivation of induced genes. In addition, Elys together with PBAP may be recruited by other dynamic Nups, such as Nup98, to the active chromatin regions located in the nuclear interior which makes these regions more favorable for transcription. These findings highlight Elys as one of the key factors participating in establishing and maintenance of genome architecture in metazoans.

## Abbreviations

CTCF	CCCTC-binding factor
Elys	embryonic large molecule derived from yolk sac
LAD	lamina-associated domain
LBR	lamin-B-receptor
MBD-R2	methyl binding domain-related 2
NSL	nonspecific lethal
NE	nuclear envelope
NL	nuclear lamina
NPC	nuclear pore complex
Nup	nucleoporin
Pc	Polycomb
PBAP	Polybromo-containing Brahma-associated proteins
TAD	Topologically associating domain
Trx	Trithorax
